# Protein Coding Low‐Copy *rpb2* and *ef1‐α* Regions Are Viable Fungal Metabarcoding DNA Markers Which Can Supplement ITS for Better Accuracy

**DOI:** 10.1002/ece3.71352

**Published:** 2025-04-21

**Authors:** Vasilii Shapkin, Miroslav Caboň, Miroslav Kolařík, Katarína Adamčíková, Petr Baldrian, Tereza Michalková, Tomáš Větrovský, Slavomír Adamčík

**Affiliations:** ^1^ Laboratory of Molecular Ecology and Mycology Institute of Botany, Plant Science and Biodiversity Center, Slovak Academy of Sciences Bratislava Slovakia; ^2^ Department of Plant Pathology University of Florida Gainesville Florida USA; ^3^ Institute of Microbiology, Academy of Sciences of the Czech Republic Prague Czech Republic; ^4^ Department of Botany, Faculty of Science Charles University Prague Czech Republic; ^5^ Department of Plant Pathology and Mycology Institute of Forest Ecology, Slovak Academy of Sciences Zvolen Nitra Slovakia; ^6^ Department of Botany, Faculty of Natural Sciences Comenius University in Bratislava Bratislava Slovakia

**Keywords:** amplicon abundance, chimera, sympatric species, threshold

## Abstract

The nuclear ribosomal DNA Internal Transcribed Spacer (ITS) region is used as a universal fungal barcode marker, but often lacks a significant DNA barcoding gap between sister taxa. Here we tested the reliability of protein coding low‐copy genes as alternative barcode markers. Mock communities of three unrelated agaric genera (*Dermoloma*, *Hodophilus*, and *Russula*) representing lineages of closely related species were sequenced by the Illumina platform targeting the ITS1, ITS2, the second largest subunit of RNA polymerase II gene (*rpb2*) and the transcription elongation factor 1‐alpha gene (*ef1‐α*) regions. Species representation and their relative abundances were similar across all tested barcode regions, despite a lower copy number in protein coding markers. ITS1 and ITS2 required more sophisticated sequence filtering because they produced a high number of chimeric sequences requiring reference‐based chimera removal and had a higher number of sequence variants per species. Although clustering of filtered ITS sequences resulted in an average higher number of correctly clustered units at optimal similarity thresholds, these thresholds varied substantially among genera. Best‐fitted thresholds of low‐copy markers were more consistent across genera but frequently lacked species resolution due to low intraspecific variability. At some thresholds, we observed multiple species lumped together, and at the same time, species split into multiple partial clusters, which should be taken into consideration when assessing the best clustering thresholds and taxonomic identity of clusters. To achieve the best taxonomic resolution and improve species detection, we recommend combining different markers and applying additional reference‐based sorting of clusters. The current availability of *rpb2* and *ef1‐α* reference sequences in public databases is far from being complete for all fungal groups, but a combined marker approach can be used for group‐specific studies that can build reference data for their own purposes.

## Graphical abstract

The study uses prepared mock communities of three unrelated basidiomycete lineages, each containing carefully selected closely related species. Performance of the generally used fungal barcoding region ITS nrDNA is compared with two low‐copy protein coding regions (rpb2 and tef1a). We demonstrate a consistent pattern across all genera, where the ITS region offers more differences among species compared to low‐copy regions, but results in a higher number of sequence variants within species and a higher number of detected chimeras requiring more difficult bioinformatic data processing. Low‐copy protein coding regions are viable markers that not only can retrieve additional species but also confirm the reliability of clustering.

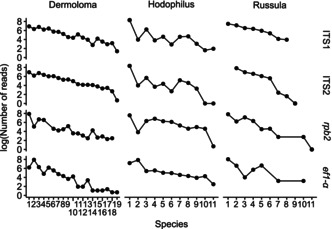

## 1 Introduction

Fungi represent a kingdom of organisms inhabiting all ecosystems and biomes on the Earth. Their presence in various substrates is usually cryptic throughout the year except for the short season when some of them (macromycetes) produce fruiting bodies. In the last decade, DNA metabarcoding became a powerful tool to detect fungal presences within various environmental samples (Nilsson et al. 2019; Tedersoo et al. 2022). This approach applies next generation high‐throughput sequencing techniques (HTS) to prepare amplicon libraries of target DNA regions (barcode marker). HTS sequencing results in thousands of sequences per sample enabling the detection of a wide range of organisms. To process the raw data and identify individual species, sophisticated bioinformatic pipelines were used (Hakimzadeh et al. 2023). After initial data quality filtering, filtered reads are clustered into operational taxonomic units (OTUs) at a defined dissimilarity threshold and compared with reference sequences of known organisms (Quince et al. 2009). Alternatively, instead of clustering sequences into OTUs, exact sequence variants (also referred to as the amplicon sequence variant) are used. This approach enhances taxonomic resolution, but it may overestimate taxon richness due to intragenomic variation among haplotypes. For this reason, the OTU‐based clustering approach is more commonly used in mycological research (Tedersoo et al. 2021).

The internal transcribed spacer (ITS) region is used as the universal fungal barcode marker (Kauserud 2023). This multicopy, noncoding ribosomal DNA region includes ITS1 and ITS2 subregions, separated by the 5.8S gene, and is situated between the small subunit (18S) and large subunit (28S) genes (Schoch et al. 2012). ITS is an obvious choice for fungal metabarcoding because of its good amplification, high mutation rate, and high representation in reference sequence databases (Bellemain et al. 2010; Nilsson et al. 2019). It has a well‐defined barcoding gap across a wide range of fungal groups, with approximately 223,659 species hypotheses clustered at the 1.5% dissimilarity threshold available in the UNITE database (Abarenkov et al. 2024). However, there are several disadvantages and limitations of the ITS region, among them the most important is the unequal number of copies among different fungal taxa ranging from 14 to more than 1000 (Lofgren et al. 2019) and high intragenomic variability related to this phenomenon (Cedeño‐Sanchez et al. 2024). This variability contributes to unequal mutation rates among different groups and low sequence conservancy, occasionally resulting in the absence of a barcoding gap (Lücking et al. 2020; Wilson et al. 2023). Furthermore, the sequence length of ITS is in some cases so variable that partial sequences can be too short or too long to overlap in the pairing step of Illumina bioinformatics (Hakimzadeh et al. 2023). Some fungal groups have poor amplification success when general fungal ITS primers are used (Tedersoo et al. 2015, 2018), which also depends on primer selection (Tedersoo and Lindahl 2016). Species detection depends on the ITS subregion choice, as some taxa are exclusively detected by either ITS1 or ITS2 (Bazzicalupo et al. 2013; Wang et al. 2015; Hoggard et al. 2018; Yang et al. 2018; Mbareche et al. 2020). In Basidiomycota, both ITS1 and ITS2 subregions show similar performances (Wang et al. 2015; Badotti et al. 2017). There is no consensus whether ITS1 or ITS2 is superior for fungal metabarcoding (Bazzicalupo et al. 2013; Yang et al. 2018) and the selection of marker may depend on the size and structure of the studied fungal community (Mello et al. 2011; Hoggard et al. 2018; Mbareche et al. 2020). Currently, both ITS subregions are used equally often (Nilsson et al. 2019; Oliveira and Azevedo 2022; Větrovský et al. 2020).

Due to the above‐described limitations of ITS as a barcode region, significant effort has been made to test alternative barcode markers for fungi. Some studies focused on other multicopy ribosomal DNA regions, such as the small and large subunits (SSU and LSU), which are less variable than ITS (Mueller et al. 2016; Lekberg et al. 2018). Currently, secondary fungal barcode markers are widely used in phylogenetic studies to complement the ITS (Xu 2016), such as protein coding regions the second largest subunit of RNA polymerase II gene (*rpb2*) and the transcription elongation factor 1‐alpha gene (*ef1‐α*). These low‐copy markers show in several cases better performance for species identification across a broad range of fungi (Matheny et al. 2007; Schoch et al. 2012; Stielow et al. 2015). Due to the wide application in fungal systematics, their representation in the NCBI database constantly grows, with over 110,000 fungal ef1‐α sequences and 67,000 for fungal rpb2 sequences available (Lücking et al. 2020). Metabarcoding targeting low‐copy regions was studied in situations where the ITS region lacks a barcoding gap to identify fungal species; for example, the *rpb1* was used to identify arbuscular mycorrhiza fungi (Stockinger et al. 2014), the *ef1‐α* for closely related *Fusarium* species (Boutigny et al. 2019) and *TUB2* for *Xylariales* (Cedeño‐Sanchez et al. 2024). Větrovský et al. (2016) compared the performance of ITS subregions and *rpb2* gene in metabarcoding of diverse fungal communities, finding that the *rpb2* gene provided higher discriminative power and better quantitative representation of amplicons among taxonomically diverse members of fungal communities in environmental samples. However, difficulty in amplifying the *rpb2* region and lack of reference sequences limit the use of this region for metabarcoding (Rué et al. 2023). Despite multiple attempts to introduce protein coding genes as alternative markers for fungal metabarcoding, their use is very rare and probably hampered by low amplification rates and lacking reference data.

To better understand PCR and sequencing biases, mock communities covering various levels of genetic variation (intragenomic, intraspecific, and interspecific) and including species classified in various taxonomic units (from genus to kingdom ranks) are created, which structure depends on the aims and character of the studies (Hleap et al. 2021). This study focused on species detection within three unrelated lineages of soil inhabiting agarics (fungi, Agaricomycotina) using different DNA markers. Underestimation resulting from pooling divergent sequences derived from related species into a single OTU can hinder our ability to recognize these species with different key roles in the ecosystem (Cristescu 2014; Conti et al. 2023). Full sympatric sister species are more common in fungi than allopatric, making their co‐occurrence quite probable (Hernández‐Hernández et al. 2021). Among the most important factors favoring sympatric ecological speciation is differentiation of resources (Thibert‐Plante and Hendry 2011; Débarre 2012). Soil heterogeneity may play a key role in promoting fungal diversity and their niche participation (Mujic et al. 2016), which underlines the ecological and potentially functional importance of distinguishing closely related species in soil samples.

**FIGURE 1:**
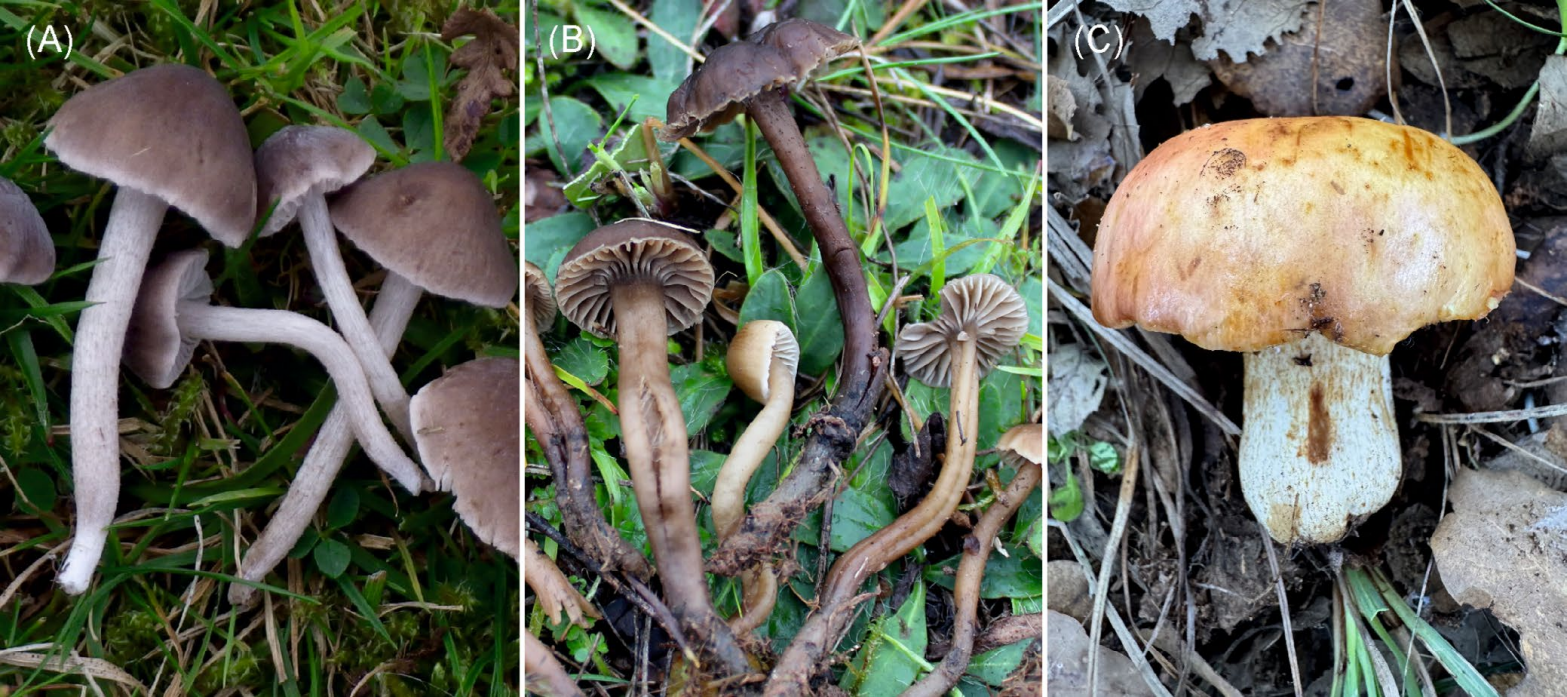
Representatives of three fungal mock communities (A–C). (A) *Dermoloma cuneifolium*. (B) *Hodophilus foetens*. (C) *Russula globispora*.

This study is the first metabarcoding performance analysis of two protein low‐copy DNA regions and two subregions of the general fungal barcode marker (ITS rDNA region) tested on fungal mock communities of three unrelated basidiomycete genera: *Dermoloma* (Tricholomataceae), *Hodophilus* (Clavariaceae), and *Russula* (Russulaceae) (Figure 1). The aim was to compare species recovery, sequencing data quality, complexity of data filtering, consistency of similarity thresholds among taxonomic groups, and application of reference data for species identification. We expect that ITS will yield a higher number of reads, but the bioinformatic processing will be more challenging, with high error rates in de novo clustering of OTUs due to unequal sequence lengths and a high number of sequence variants. Low‐copy genes may suffer from low read yields due to low amplification success, but we expect that they will retrieve species not detected by ITS and a more homogenous representation of reads among species.

## 2 Materials and Methods

### 2.1 Mock Community Sampling and Reference Data

Each selected model fungal agaric genus was represented by evolutionary related species which show low genetic distances in several cases. 19 species of the genus *Dermoloma* (Tricholomataceae), 11 of *Hodophilus* (Clavariaceae) and 11 of *Russula* subsect. *Maculatinae* (Russulaceae) were selected. All species represented by a single DNA sample were provided with Sanger‐based sequence references of internal transcribed spacer region ITS1‐5.8S‐ITS2 (ITS), a portion of the *ef1‐α* gene, and the 5th partition of the *rpb2* gene. Most of ITS sequences and part of *Russula ef1‐α* sequences were available from parallel phylogenetic studies (Adamčík et al. 2016, 2017, 2018, 2020; Caboň et al. 2019; Kiran et al. 2021; Sánchez‐García and Matheny 2017; Sánchez‐García et al. 2021; Vidal et al. 2019). We used DNA gathered during these studies to sequence missing references by the Sanger method for the purpose of this study. Because 5th partition used in this study was not covered by published *rpb2* sequences, the reference sequences of all samples were *de novo* sequenced. *Dermoloma* cf. *pseudocuneifolium* and *D*. sp. 6 are provided by new unpublished samples due to the low quality of DNA of samples of these species included in the previous study (Sánchez‐García et al. 2021). For the same reason, we used unpublished samples of *R. dryadicola* and *R. globispora* in addition to five other samples of so far unpublished *Russula* species. All used samples and accession numbers of corresponding reference sequences are listed in Table S1. Newly generated sequences are deposited in Genbank. Our reference dataset is almost complete, with the exception of three missing *rpb2* sequences that failed to amplify. Primer pairs and PCR conditions used for Sanger sequencing are listed in Table S2.

### 2.2 Mock Community Construction and Amplicon Sequencing

DNA samples of individual species were quality checked with a Shimadzu BioSpec‐nano spectrophotometer (Shimadzu, Japan) and quantified using a Thermo Fisher Qubit Fluorometer dsDNA HS Assay Kit (Thermo Fisher, USA). Mock communities were assembled for each genus separately by pooling all samples at equal DNA proportions to the final concentration of 5 ng/μL. For every tested molecular marker (ITS1, ITS2, *rpb2*, and *ef1‐α*), PCR amplifications of each mock community were performed in triplicates following the protocols and conditions explained in Table S3. The quality and yield of PCRs were visually confirmed on a 1% agarose gel; then, PCR triplicates were pooled together, purified using the QIAquick PCR Purification Kit (Qiagen, Hilden, Germany), and quantified using a Thermo Fisher Qubit Fluorometer dsDNA HS Assay Kit (Thermo Fisher, USA). The amplicon library was prepared using a TruSeq DNA PCR‐free Kit (Illumina, San Diego, CA, USA) and sequenced in triplicates on an Illumina MiSeq platform using the MiSeq Reagent Kit v2 for 2 × 250 bp reads at the Institute of Microbiology of the CAS.

### 2.3 Sequence Data Processing

Illumina sequencing yielded 740,578 paired‐end reads with a 92.5% proportion of high quality (Phred quality score above 30) bases. USEARCH version 11.0.667 (Edgar 2010) was used to process sequence data. R1 and R2 reads were paired using the *fastq_mergepairs* command specifying the following parameters: *‐fastq_nostagger ‐fastq_minovlen* 25 *‐fastq_maxdiffs* 0. Primer sequences were trimmed at both ends using the *fasta_truncate* command. Subsequently, reads were quality filtered based on the maximum error (maxee) probability using the *fasta_filter* command with options *‐fastq_maxee* 1 *‐fastq_minlen* 50 (ITS1 and ITS2)/200 (*rpb2* and *ef1‐α*). Conservative flanking parts of ITS1 and ITS2 reads were trimmed using the ITSx (Bengtsson‐Palme et al. 2013) fungal extractor. Chimeric reads were detected and removed using the UCHIME (Edgar et al. 2011) algorithm implemented in the following USEARCHs commands: *uchime3_denovo* for de novo search and *uchime2_ref* (sensitive mode) for a reference‐based search. Nontarget reads, which could not be confidently assigned to any sequence in a reference dataset, were discarded. The numbers of reads sorted by each filtering step are listed in Table S4. Discarded reads were assigned to genus rank units using a BLASTn search with a best hit of at least 90% similarity to a sequence in the NCBI database with a minimum query coverage of 95%.

### 2.4 Taxonomic Assignment

Filtered Illumina reads were annotated against reference sequences using USEARCH's *annot* command, applying a 97% similarity threshold. Based on Sanger reference sequences, distinguishing positions of each species within each barcode marker were identified. For each individual species, all annotated reads of each tested barcode region were manually aligned with reference Sanger sequences. Identifications of all Illumina amplicon variants were verified by comparing distinguishing positions of reference sequences within this alignment. Ambiguities in Sanger reference sequences matching differences in amplicon sequences were used to identify infraspecific variability and are recognized as sequence variants of individual species. Any other differences within the amplicon dataset of a single species not matching reference sequences are treated as sequencing noise. Closely related species not showing significant differences in some barcode regions are labeled as a single unit for the purpose of analyses. Species that did not appear in the dataset after taxonomic assignment were manually traced among unmerged and chimeric reads.

### 2.5 OTU Clustering

Labeled reads were clustered into operational taxonomic units (OTUs) at a gradually increasing similarity threshold starting from 95% to 99.5% with a 0.5% step using the UCLUST (Edgar 2010) algorithm implemented in USEARCH's *cluster_fast* command. For each clustering step, we analyzed the number of clusters produced and the distribution of annotated reads among them. OTUs that included all reads of a single species (and no other species reads) were recognized as correct matches, OTUs grouping part of a single species reads as partial matches, and the ones grouping reads from two or more species as mixed. Partial or mixed OTUs were counted as mistakes; more species clustered into a single OTU were counted as multiple mistakes.

### 2.6 Final Statistical Analyses

Reads were randomly resampled to the same sample depth of 5000 reads using USEARCH's *fastx_subsample* command. All data were imported into R version 4.2.0 (R Core Team 2023) for statistical analyses and visualization. One‐way analysis of variance (one‐way ANOVA) was performed for the comparison of read distribution among species in different barcoding markers. Spearman's rank correlation (*ρ*) was used to determine correlation between relative abundance of individual species and mistake rate. The data were logarithmically transformed to meet normality and statistically analyzed using *dplyr* version 1.1.4 (Wickham et al. 2023) and *rstatix* version 0.7.2 packages (Kassambara 2023). All graphics were generated using *ggplot2* version 3.4.4 (Wickham 2016).

## 3 Results

### 3.1 Initial Sequence Filtering

**FIGURE 2:**
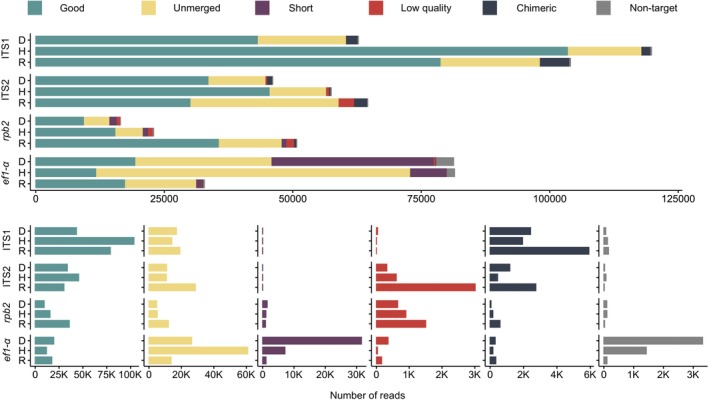
Sequence filtering results in mock communities of three fungal genera sequenced by Illumina MiSeq using four barcode regions. D—*Dermoloma*, H—*Hodophilus*, R—*Russula*. Short reads are merged reads shorter than 50 bp for ITS1 and ITS2 regions and shorter than 200 bp for protein coding regions. Chimeric reads are detected by a reference‐based approach. Nontarget reads represent good quality merged reads that passed initial data filtering but do not match the reference dataset.

The total high‐throughput sequencing depth across all three genera was 286,630 for ITS1, 168,192 for ITS2, 195,578 for *ef1‐α*, and 90,178 for *rpb2* (Figure 2). The highest sequencing depth was observed in *Dermoloma* for the *ef1‐α* region, whereas the other two genera exhibited the greatest depth in the ITS1 region. However, during the filtering steps, a large number of reads were discarded from the *ef1‐α* datasets (75%) mainly due to unmerged sequences. There was a high proportion of merged but short sequences discarded in *Dermoloma* and, to a lesser extent, also in *Hodophilus*.

Low quality‐merged sequences and chimeric sequences were less represented than unmerged and too short reads. Low‐quality sequences were more frequent in ITS2 and *rpb2* regions and especially in the genus *Russula*. Chimeric reads were more frequent in both regions of ITS and particularly in *Russula*. UCHIME's de novo algorithm detected 78% of chimeras detected using the reference database in ITS1, 89% in ITS2, 60% in *rpb2*, and 81% in *ef1‐α*. Although undetected reads in ITS1 and ITS2 were mainly chimeras with low similarity to reference sequences (< 97%), in rpb2 and ef1‐*α* these reads could not be matched to any reference sequences. The BLASTn search of all discarded reads against the NCBI database showed that 43% of *ef1‐α* reads and 45% of *rpb2* were nontarget sequences with no similarity to any sequence in the reference database; the proportion of such reads in ITS1 and ITS2 was < 0.05%. A small proportion of nontarget reads passed the initial filtering steps, of which a notably higher number (2.5%) of such reads was detected in *ef1‐α* (Table S4).

After quality filtering, ITS1 had the highest number of reads across all three genera. For *Hodophilus* and *Russula*, ITS1 yielded more than twice as many reads as any other tested region. Most of the filtered libraries of low‐copy regions yielded fewer reads than ITS multicopy regions, except in *Russula*, which had more reads in *rpb2* than in ITS2.

### 3.2 Species Recovery

*Hodophilus* was the only genus where all tested barcode regions recovered all species (Table 1). However, the ITS2 reads for *H. pallidus* and *H. phaeophyllus* were below the threshold of 0.01% commonly applied to accept OTU presence in metabarcoding studies. In *Dermoloma*, *D*. cf. *pusillum* and *D*. cf. *cuneifolium* USA were absent in the *rpb2* dataset, and the latter species was below the 0.01% threshold in ITS2 and *ef1‐α* datasets. In *Russula*, there was the highest number of undetected species. The best result showed low‐copy regions; only one of 11 species was missing in *rpb2* and two were missing in *ef1‐α*. However, one species pair in *rpb2* and two in the *ef1‐α* region could not be differentiated, and each such pair was recognized as a single unit. Three species were not detected in each of the ITS1 and ITS2 regions, and the presence of *R. abbotabadensis* in ITS2 was below the 0.01% threshold. The majority of absent species or species below the 0.01% threshold are likely due to degraded DNA. The rpb2 region of *D. cf. pusillum* was neither obtained by Sanger sequencing nor detected in the metabarcoding dataset, strongly suggesting primer incompatibility. *Russula* sp. 1 was absent in the filtered ITS2 dataset but had the highest number of reads in all other regions. The ITS2 region for this species was too long to be fully covered with two paired‐end reads, although 17,251 single unmerged reads corresponding to this species were found among discarded sequences.

**TABLE 1:** Species representation in mock communities of metabarcoding datasets of four DNA regions sequenced by Illumina MiSeq platform. The species are arranged in descending order by total count of sequence representation within all four regions.

#	Species	ITS1		ITS2		*rpb2*		*ef1‐α*	
		RA	SV	RA	SV	RA	SV	RA	SV
*Dermoloma*									
1	sp. 8	1002	1	1031	1	2682	1	496	2
2	*bellerianum*	555	2	486	2	162	2	2751	1
3	*phaeopodium*	884	3	854	3	798	1	541	3
4	sp. 6	495	1	576	1	699	2	122	2
5	cf. *pusillum*	627	2	418	2	—	—	479	2
6	*cuneifolium*	314	1	428	1	99	1	257	2
7	cf. *cuneifolium* 1	298	1	284	1	68	1	114	2
8	sp. 3	186	2	196	2	88	1	74	1
9	*pseudocuneifolium*	93	1	206	2	176	1	39	1
10	*alexandri*	80	1	142	2	36	1	67	1
11	sp. 11	169	2	72	1	35	1	7	1
12	cf. *pseudocuneifolium*	82	2	63	1	22	1	7	1
13	cf. *phaeopodium*	52	2	63	2	12	1	29	2
14	sp. 4	16	1	60	1	69	1	3	2
15	*magicum*	66	1	44	1	14	1	3	1
16	cf. *cuneifolium* 2	34	1	28	1	18	1	3	2
17	*atrocinereum*	19	1	32	2	10	1	4	1
18	sp. 2	24	2	15	2	12	1	2	1
19	cf. *cuneifolium* USA	4	1	2	1	—	—	2	1
*Hodophilus*									
1	*foetens*	4034	2	4219	1	1949	1	1313	1
2	*atropunctus*	53	2	55	2	46	1	2609	2
3	*carpaticus*	508	1	293	1	554	1	223	1
4	*micaceus*	45	1	41	1	918	1	256	1
5	*phaeoxanthus*	101	2	79	1	527	1	154	2
6	*cambriensis*	18	1	15	1	455	1	142	1
7	*anatinus*	99	2	173	2	205	1	95	2
8	*variabilipes*	109	1	96	1	101	1	75	2
9	*stramineus*	21	1	27	2	144	2	50	1
10	*pallidus*	5	1	1	1	99	1	71	2
11	*phaeophyllus*	7	1	1	1	2	2	12	1
*Russula*									
1	sp. 1	1767	2	–^a^	—	2480	1	3094	1
2	sp. 2	1260	1	2525	1	500	1	732	1
3	sp. 3	684	1	1031	1	1240	2	55	1
4	*dryadicola*	583	2	727	2	557	2	302	1
5	sp. 4	378	1	434	2	90	1	767	1
6	*mattiroloana*	214	1	266	1	100	1		
7	sp. 5	62	1	11	1	16	1	25	1
8	*globispora*	52	2	5	1				
9	*quercus‐floribundae*	—	—	—	—	16	1	25	1
10	*abbottabadensis*	—	—	1	1	—	—	—	—
11	*ayubiana*	—	—	—	—	1	1	—	—

Abbreviations: RA, relative abundance of sequences; SV, number of sequence variants.

^a^ ITS2 reads of *Russula* sp. 1 were filtered out during the pairing step because they were too long.

### 3.3 Relative Abundance

Multiple sequence variants were present in 36% of species in ITS1, 34% in ITS2, 34% in *ef1‐α*, and 15% in the *rpb2* region. The number of polymorphic positions within a single species varied from 1 to 4 in multicopy genes and 1 to 2 in low‐copy genes for most species, with few exceptions. *D. bellerianum* showed an unusually high degree of polymorphism with 16 polymorphic positions in the ITS2 region and 7 in ITS1; however, there were only three polymorphic positions in *rpb2* and none in the *ef1‐α* region. A high degree of polymorphism was also observed in the ITS2 region of *H. atropunctus* (6 positions), the ITS1 region of *R. globispora* (5 positions), and the *ef1‐α* region of *D. cf. cuneifolium* 1 (4 positions).

**FIGURE 3:**
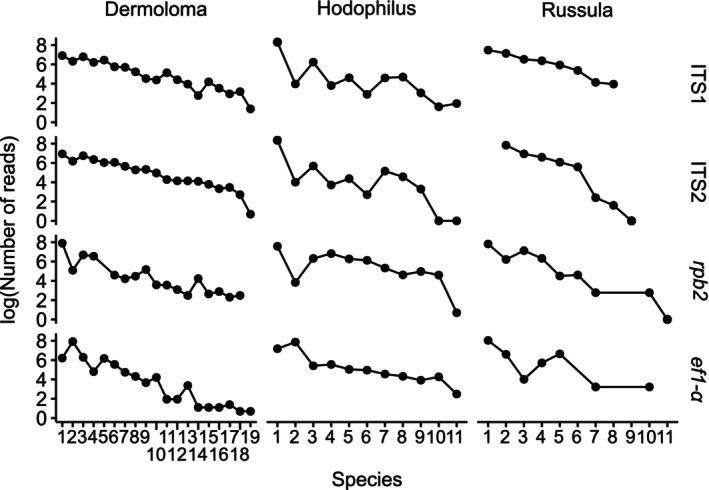
Logarithmic read distribution of the detected species among mock communities of three fungal genera sorted by total count of reads within four barcode regions. Numbers on the *x*‐axis correspond to the species order in Table 1.

The general pattern of reads distribution was similar across the tested DNA genes for each region (Figure 3). ANOVA test showed no significant differences in read distribution among individual barcode regions within each genus (*p* = 0.134 for *Dermoloma*, *p* = 0.275 for *Hodophilus*; *p* = 0.648 for *Russula*). Several species displayed contrasting abundances across different DNA regions, for example, *D*. sp. *8*, *D. bellerianum*, *H. foetens*, *H. atropunctus*, *H. micaceus*, *R*. sp. 2, and *R*. sp. 3. The best‐balanced curve with the least number of changes in species order of relative abundance for *Dermoloma* showed ITS2, for *Hodophilus ef1‐α* and for *Russula* ITS1. Low‐copy protein coding genes (*rpb2* and *ef1‐α*) did not show better balance between relative abundance of individual species compared to multicopy regions (ITS1 and ITS2).

### 3.4 OTU Clustering

**FIGURE 4:**
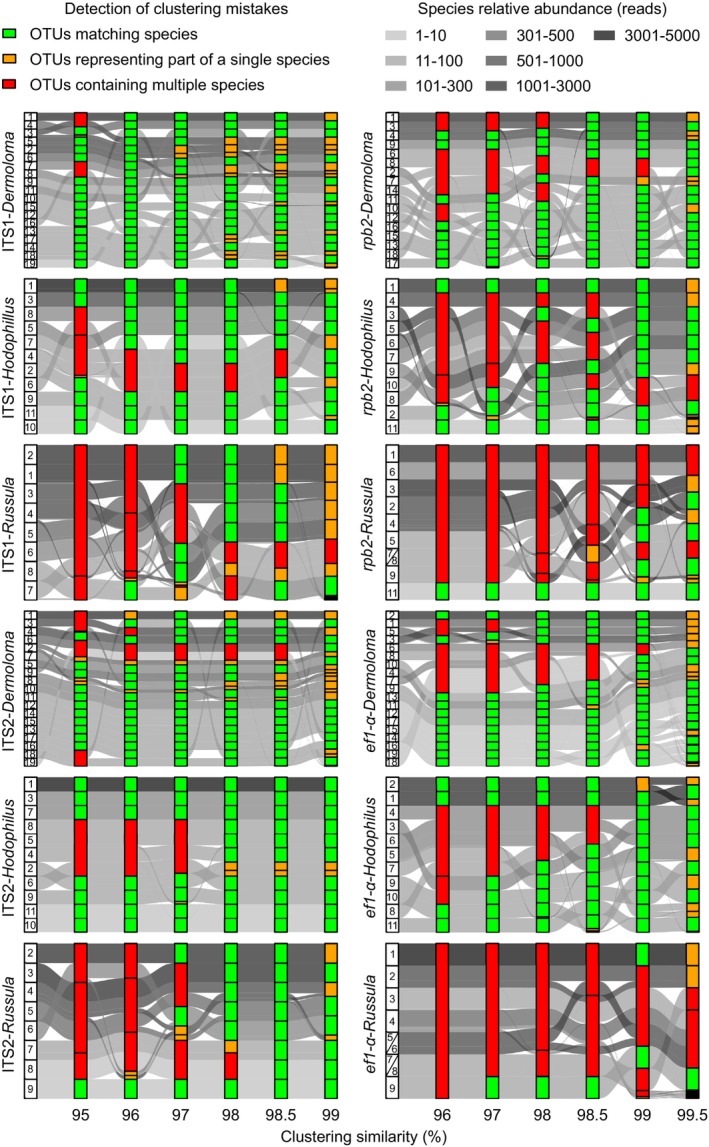
Species clustering in OTUs for different combinations of mock communities, barcode regions and similarity thresholds. Multiple species clustered in a single OTU and OTUs with part of a single species reads are counted as mistakes. Black bars present for example in 99.5% of *ef1‐α* in *Russula* are accumulations of small OTUs. More details about species relative abundance are in Table 1.

Automated USEARCH clustering resulted in a very different combination of best‐performing barcode markers and clustering similarity thresholds for different mock communities (Figure 4). For *Dermoloma*, only ITS1 clustered at a 96% similarity threshold perfectly matched all 19 phylogenetic species in the mock community. The highest number of correctly clustered OTUs in *Hodophilus* was 10 of 11, and this was generated by 98%, 98.5%, and 99% similarity thresholds applied on ITS2 and 99% threshold applied on *ef1‐α*. The best combination for *Russula* performed best with ITS2 at a 98.5% threshold, which correctly identified 8 of 11 species. However, *Russula* sp. 1, the most abundant OTU, was only correctly detected by ITS1 (97% and 98%) and ef1‐α (99%).

Spearman's rank correlation did not reveal a significant overall positive correlation between the relative abundance of individual species and the mistake rate (*ρ* = 0.453, *p* < 0.005). A significantly higher mistake rate was also observed for species with a higher number of sequence variants (*ρ* = 0.326, *p* < 0.005).

**FIGURE 5:**
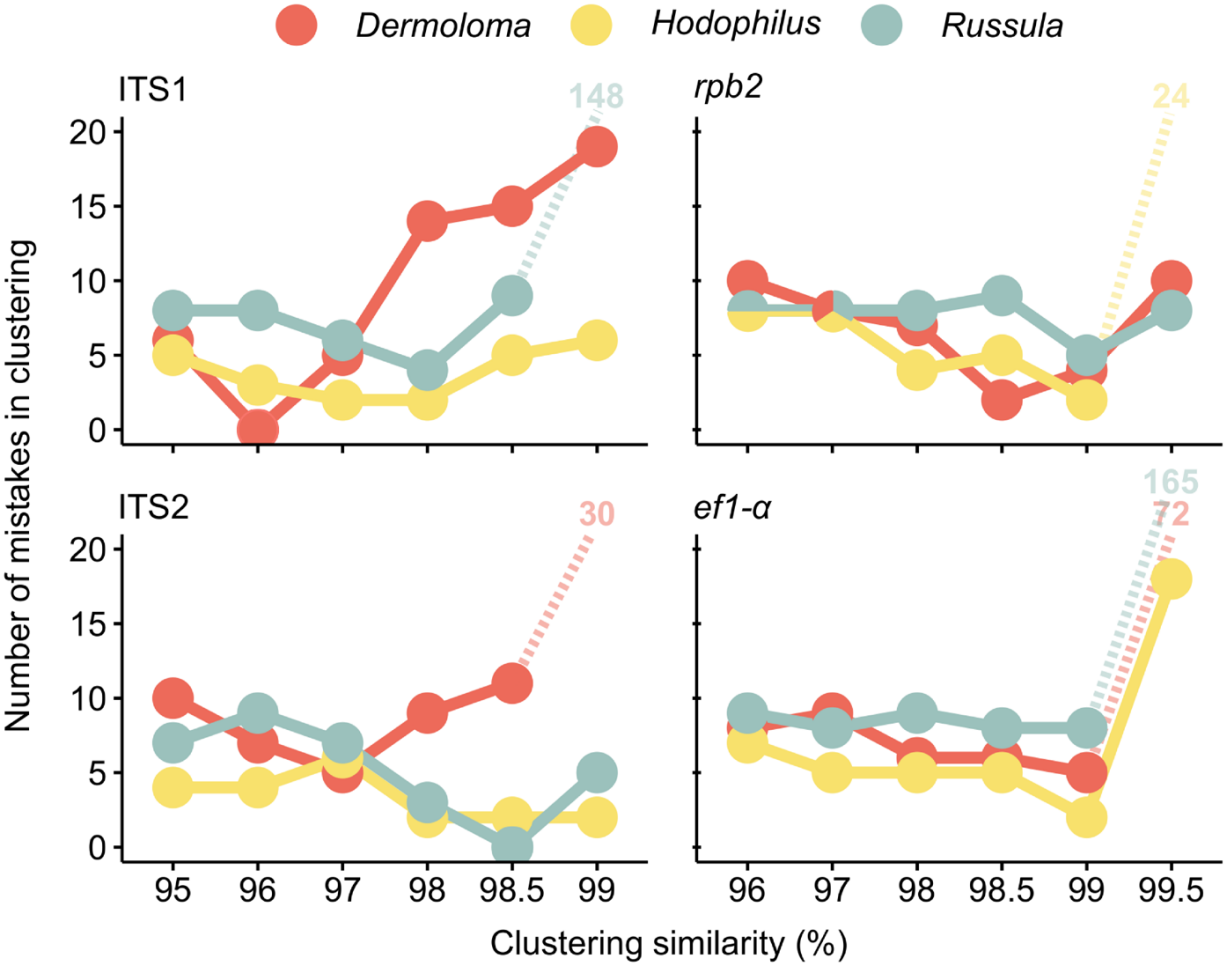
Comparison of mistakes at different similarity thresholds for three mock communities and four barcode regions. Multiple species clustered in a single OTU and OTUs with part of a single species reads are counted as mistakes.

The only situation when all three mock communities showed identical best results was ef1‐α clustering at a 99% similarity threshold (Figure 5). The best clustering thresholds of the other three regions were similar for *Russula* and *Hodophilus* but differed for *Dermoloma*. Specifically, ITS1 performed best at a 98% threshold for *Hodophilus* and *Russula* but at a 96% threshold for *Dermoloma*. For ITS2, the best result was 98.5% for *Hodophilus* and *Russula* and 98.5% for *Dermoloma*. Best results of *rpb2* were at a 99% threshold for *Hodophilus* and *Russula* and 98.5% for *Dermoloma*.

### 3.5 General Comparison of Barcode Marker Performances

There was no single barcode marker that clearly outperformed others across all parameters. Illumina sequencing of both ITS1 and ITS2 resulted in higher sequence depth before and after initial filtering. In this parameter, *ef1‐α* had the lowest sequence depth and highest number of discarded reads, especially because of unmerged and short reads. On the other hand, both ITS regions suffered from a higher presence of chimeric sequences, the detection of which depended on the reference database. All four regions recovered approximately the same number of species, with a few species lost mainly due to a low DNA quality or absence of a barcoding gap, but in a few cases, also primer incompatibility and too long barcode region. However, the limiting factor for assessing the expected species richness in metabarcoding analysis is the ability to correctly recognize OTUs without the presence of partial or mixed OTUs contributing to overestimating or underestimating. There was an imbalance among mock communities of individual genera for each barcode marker, showing various proportions of correctly recovered species at the best threshold. However, these best thresholds differed among mock communities, with only *ef1‐α* providing a single best‐performing threshold consistently across all three genera.

## 4 Discussion

### 4.1 Sequencing Depth and Quality

Shorter barcode regions are amplified more efficiently, yielding higher sequencing depth in Illumina sequencing (Castaño et al. 2020). This observation agrees with our results, as demonstrated by the ITS1 region of *Hodophilus*, which was shorter than 200 bp long and yielded the highest sequencing depth (Figure 2). Longer sequences experience a decline in base call quality at the 3′ end, causing merging failures during the bioinformatics (Tan et al. 2019). We observed a high proportion of low‐quality reads filtered out from datasets with longer barcode sequences, for example, *rpb2* of all three genera (ranging in average length of 395–400 bp) and ITS2 of *Russula* (average length 405 bp). Větrovský et al. (2016) reported *rpb2* performance similar to ITS region, which does not agree with the low sequencing depth of this region observed in this study. However, they used 454 pyrosequencing applied on the longer 6th partition of *rpb2*, and in this study, we used primers for the shorter 5th partition because we used the Illumina platform. Use of the 6th partition on the Illumina platform would probably lead to much lower *rpb2* performance, as reported by Rué et al. (2023). The relatively good species recovery rate, which was consistent across all three genera at a 99% threshold, indicates that *rpb2* is a reliable metabarcoding primer. Uneven sequencing depth, which is almost equal to ITS in *Russula*, but lower in the other two genera, suggests that the design of *rpb2* primers and sequencing conditions need further optimization to cover more efficiently other fungal groups. Only in the case of *R*. sp. 1 with ITS2 of ca. 465 bp long, the species was not recovered due to failure of merging reverse and forward reads, but the species was detected as abundant in discarded unmerged reads. This length heterogeneity in the ITS region is well known (Feibelman et al. 1994; Bellemain et al. 2010) and, for example, it includes insertions of homologous, coding, up to 255 bp long, Signal Recognition Particle in the noncoding part of ITS1 of several species of unrelated ectomycorrhizal genera, including also some *Russula* species (Rosenblad et al. 2016).

The highest chimera detection rate occurred in the ITS1 region (on average 3.7%), exceeding the rate for ITS2 (average 2.5%) across all three genera. Both ITS1 and ITS2 regions had a higher proportion of detected chimeras compared to protein coding genes (0.5% for *tef1‐α* and 0.7% for *rpb2*). The high rate of chimera formation is well known for rDNA barcode regions, but among them, ITS2 has a lower rate compared to 16S and 18S markers (Aas et al. 2017). The chimera formation rate increases with the level of sequence relatedness (Shin et al. 2014; Aas et al. 2017) as the polymerase enzyme more easily combines similar templates. Our dataset was primarily designed to include phylogenetically related species; thus, we expected a higher proportion of chimeras compared to other metabarcoding studies. It is well supported in our study that ITS has a much higher chimera rate than *tef1‐α* and *rpb2*, despite closer genetic distances between species in the protein coding region. A lower proportion of chimeras detected for low‐copy regions in our study agrees with the finding of Větrovský et al. (2016) for *rpb2* and Trollip et al. (2024) for *ef1‐α*. The chimera detection rate of protein coding markers in our study was lower, despite our applying a higher number of PCR cycles during the amplification of these markers (Table S3), which is known to increase chimera presence (Bakker 2018; Sze and Schloss 2019). The mechanism standing behind the observed differences is unknown, though higher infraspecific sequence variability and a higher number of sequence variants in ITS may contribute to this. Incomplete chimera removal may lead to OTU diversity overestimation, and therefore, higher chimera formation is more challenging for bioinformatics (Abarenkov et al. 2024). Although some studies suggest that the de novo chimera detection approach outperforms the reference‐based approach (Aas et al. 2017; Schloss et al. 2011; Edgar et al. 2011), we detected more than 30% of chimeras that were not detected by the first approach, highlighting the importance of robust reference databases (Nilsson et al. 2015). Manual examination of all discarded reads confirmed that none of the initially included mock community species were mistakenly removed as chimeric reads, suggesting minimal negative effects from potential false‐positive chimeras (Tedersoo et al. 2021). Relying strictly on the ITS region that produces a high rate of chimeric reads may lead, without their removal, to false OTUs. Chimera detection requires the implementation of more challenging bioinformatic analyses, which may consequently lead to imprecise estimation of species richness (Pauvert et al. 2019; Hakimzadeh et al. 2023). Combining ITS with low‐copy markers can mitigate negative effects linked to the high presence of chimera in ITS.

Advantage of protein coding markers is their low chimera frequency, but their disadvantage is lower sequencing depth, partly due to a higher proportion of discarded reads resulting from amplification of nontarget DNA. In our mock communities, the proportion of discarded reads ranged from 30% to 43% for *rpb2* and 47% to 86% for *ef1‐α*. This proportion of discarded *rpb2* reads was comparable with the performance of ITS markers in our dataset. Větrovský et al. (2016) obtained an even higher proportion of good *rpb2* reads compared to ITS when they tested a mock community of random fungi from different phyla, but they used only 17% of *rpb2* filtered reads from tested environmental samples, indicating that sample source and character can have an impact on the quality of sequencing. The proportion of filtered good *ef1‐α* reads observed in the *Fusarium* mock community (2.3%) and environmental samples (2.4%) was even lower than in our study (Boutigny et al. 2019). This suggests that the quality of sequencing is influenced by multiple factors such as sample source, community composition, DNA extraction methods, polymerase, PCR conditions, and primer degeneration (Bakker 2018; van der Loos and Nijland 2021; Tedersoo et al. 2022). Low primer specificity might explain the low sequencing depth and high proportion of nontarget reads in *rpb2*. Using a multiplex of primers for a single marker may improve PCR amplification and species coverage, but requires methodological optimization and represents a bioinformatic challenge (Ficetola and Taberlet 2023).

### 4.2 Species Recovery and Relative Abundance

Using alignment‐based taxonomic assignment including the checking of distinguishing positions recognized in reference datasets resulted in confident recovery of the majority of species (73%) across datasets sequenced by all four barcode markers (Table 1). All *Hodophilus* species were recovered by all markers. In *Dermoloma*, two species were not recovered by *rpb2*, and in the case of one of them (*D*. cf. *pusillum*) it is apparently due to primer incompatibility because also Sanger sequencing of this species failed, while sequencing results of other barcode markers were good. Only three of 11 *Russula* species were consistently recovered by all four regions. One species was missing due to too long ITS2 sequences, as mentioned above. Two *Russula* species pairs were not recovered by low‐copy regions due to the absence of differences in short ca. 400 bp long Illumina reads, but differences among species were identified in longer partitions of these regions (unpublished data). Our sampling shows different evolutionary rates observed in various lineages of Agaricomycetes (Varga et al. 2019), with the genus *Russula* representing a lineage with rapid evolutionary radiation known especially for species lineages in temperate and boreal areas of the Northern Hemisphere (Hackel et al. 2022). Low number of reads or absence of some species in one or more barcode regions is apparently due to partial DNA degradation, as suggested by the bad quality of electrophoretograms or amplification failures observed during Sanger sequencing. Examples of this possible DNA degradation are *D*. cf. *cuneifolium* USA, *H. phaeophyllus*, *R. abbottabadensis*, *R. ayubiana*, and *R. quercus‐floribundae*. Occasionally, low‐copy regions provide better sequence representation than ITS, for example, in *R. quercus‐floribunae* and *H. pallidus* . This shows a perspective of low‐copy regions for better species recovery of low‐represented species with significant ecological function, which presence in the sample depends on seasonality, water dynamics, or trophic interactions (Abu Almakarem et al. 2012; Matsuoka et al. 2021; Schmidt et al. 2018).

The pattern in descending order of relative abundance is similar for all mock communities and markers in our experiment (Figure 3), with occasional differences in amplicon proportion per species. Contrary to Větrovský et al. (2016), we did not find *rpb2* superior in the estimations of diversity and relative abundance. Instead, our results align with Ogier et al. (2019), indicating that low‐copy genes are not more reliable for the estimation of relative abundances. This study provides more insight into biases in relative read abundance that are important for the estimation of diversity in metabarcoding studies (Deagle et al. 2019; van der Loos and Nijland 2021; Mächler et al. 2021). Mock communities tested in this study were exclusively composed of lineages of closely related species, but according to some studies, species abundances would not be influenced by sample complexity and other species presence (Bell et al. 2019).

### 4.3 OTU Clustering

To process complex data in metabarcoding analyses, after initial data filtering, reads are clustered into OTUs, which are finally identified to taxa at various taxonomic ranks using reference databases (Hakimzadeh et al. 2023). Reliable taxonomic assignments depend on clustering bioinformatics and the existence of a barcoding gap with differences between species verified by a coalescent approach (Ryberg 2015). To avoid incorrect taxonomic assignment for OTUs, it is important to employ taxon‐specific distance thresholds (Phillips et al. 2022). Several studies recommended careful setup of similarity clustering thresholds considering research objectives, phylogenetic diversity of the studied group, required taxonomic accuracy, and potential oversplitting and overmerging (Voyron et al. 2017; Bonin et al. 2023; Wilson et al. 2023). Our study showed that most combinations of mock communities and barcode markers resulted in the merging of multiple species into mixed OTUs combined with the presence of incomplete OTUs (which contain part of reads of a single species but not all of them). This means that at some thresholds, diversity estimation errors occur despite OTU counts matching expected species numbers. While such errors may minimally impact diversity indices, they pose significant problems for studies depending on correct species identification, for example, in case of ecological studies focused on defined trophic, ecological, or taxonomic groups where ecological profiles of OTUs are estimated based on matches with the known ecology of reference species (Keck et al. 2018). Our study indicates the importance of well‐defined species delimitations in the reference database that may be used as part of a control to avoid false species presence data (both negatives and positives) by filtering of clustering criteria and adjusting thresholds (Zinger et al. 2019). In case of taxonomic incongruence with a priori expectation, identifications of OTUs assigned to the best match in the reference database by the bioinformatic pipeline should be verified by a phylogenetic approach using alignment‐based tree reconstruction (Hleap et al. 2021).

## 5 Conclusions

All four barcode markers tested here have their advantages and limitations. For studies where reference data exist also for protein coding regions, it is worth considering sequencing a pair of a multicopy and a low‐copy region for the identification of missing or incorrectly clustered OTUs and for confirmation of species presences. Implementation of low‐copy regions might be especially applicable in taxon or group‐specific studies, in such disciplines as the food industry, environmental monitoring, or wildlife forensics (Arulandhu et al. 2017). For such studies, designing group‐specific primers can mitigate the problem with the low PCR amplification rate of *rpb2* and *ef1‐α*. To simplify sequence identification among different barcoding regions, it will be important to link fungal ITS data with other barcoding regions in such databases as UNITE (Abarenkov et al. 2024) and GlobalFungi (Větrovský et al. 2020). Different species recovered in our study by different barcode markers confirmed the good‐practice recommendation to combine widely used molecular markers with an additional one for higher taxonomic resolution within individual groups of organisms (Lekberg et al. 2018; van der Loos and Nijland 2021; Ogier et al. 2019; Swenie et al. 2024). This study showcases the advantage of the combined metabarcoding markers approach on the example of three Basidiomycota lineages which cover a significant phylogenetic variation within Agaricomycotina, but future testing on a broader representation of other fungal lineages could strengthen the conclusions.

## Author Contributions

**Vasilii Shapkin:** data curation (equal), formal analysis (equal), methodology (equal), software (equal), visualization (equal), writing – original draft (equal). **Miroslav Caboň:** data curation (equal), formal analysis (equal), validation (equal), writing – review and editing (equal). **Miroslav Kolařík:** conceptualization (equal), validation (equal), writing – review and editing (equal). **Katarína Adamčíková:** data curation (equal), writing – review and editing (equal). **Petr Baldrian:** funding acquisition (equal), supervision (equal), writing – review and editing (equal). **Tereza Michalková:** formal analysis (equal), writing – review and editing (equal). **Tomáš Větrovský:** software (equal), validation (equal), writing – review and editing (equal). **Slavomír Adamčík:** conceptualization (equal), funding acquisition (equal), methodology (equal), resources (equal), supervision (equal), writing – original draft (equal), writing – review and editing (equal).

## Disclosure

Benefit‐Sharing Statement: The majority of fungal genomic DNA used in this study is deposited in Plant Science and Biodiversity Centre SAS and was gathered from parallel published phylogenetic studies (Table S1). Almost all new, previously unpublished fungal samples were collected by authors of this study and do not contain material regulated by the Nagoya protocol or any other restrictions of local governments. One fungal collection was loaned from a public herbarium KRA‐M, and the DNA was extracted and used under permission of the curator.

## Conflicts of Interest

The authors declare no conflicts of interest.

## Supporting information


Appendix S1

## Data Availability

The manuscript does not use ecological metadata but DNA from another study. Ecological metadata will be accessible at the NCBI database where the used sequences are deposited. Raw sequence Illumina reads are deposited in the SRA (BioProject PRJNA1249409). Sanger‐generated reference sequences produced in this study are deposited to NCBI Nucleotide Database and are listed in Table S1.
