# Correction to “Protein Coding Low‐Copy *rpb2* and *ef1‐α* Regions Are Viable Fungal Metabarcoding DNA Markers Which Can Supplement ITS for Better Accuracy”

**DOI:** 10.1002/ece3.71598

**Published:** 2025-06-30

Shapkin, V., Caboň M., Kolařík, M., Adamčíková, K., Baldrian, P., Michalová, T., Větrovský, T. and Adamčík, S. (2025), Protein Coding Low‐Copy *rpb2* and *ef1‐α* Regions Are Viable Fungal Metabarcoding DNA Markers Which Can Supplement ITS for Better Accuracy. Ecol Evol, 15:e71352. https://doi.org/10.1002/ece3.71352

The spelling of surname of the author “Tereza Michalková” was incorrect. This should have read: “Tereza Michalová”. The online version of this article has been corrected accordingly.

We apologize for this error.

